# A dose–response meta-analysis between serum concentration of 25-hydroxy vitamin D and risk of type 1 diabetes mellitus

**DOI:** 10.1038/s41430-020-00813-1

**Published:** 2020-11-24

**Authors:** Yilin Hou, An Song, Yuxin Jin, Qiuyang Xia, Guangyao Song, Xiaoping Xing

**Affiliations:** 1 Department of Internal Medicine, Hebei Medical University, Shijiazhuang, 050017 Hebei PR China; 2 Endocrinology Department, Hebei General Hospital, Shijiazhuang, 050051 Hebei PR China; 3 Key Laboratory of Endocrinology, Ministry of Health, Department of Endocrinology, Peking Union Medical College Hospital, Peking Union Medical College, Chinese Academy of Medical Sciences, 100730 Beijing, PR China

**Keywords:** Type 1 diabetes, Insulin-dependent diabetes, Vitamin D, 25-hydroxy vitamin D, Meta-analysis, Dose response, Type 1 diabetes, Risk factors

## Abstract

It remains debatable whether vitamin D plays any role as a risk factor for type 1 diabetes mellitus (T1DM). We have summarized the effect of circulating 25-hydroxy vitamin D [25(OH)D] concentration on the risk of developing T1DM via a dose–response meta-analysis. We undertook a database search on PubMed, Embase, and Cochrane Library from inception to January 2020. A meta-analysis based on random-effects model was applied. Subgroup analysis and meta-regression were performed to inspect the source of heterogeneity. Dose–response data were examined using the generalized least squares trend estimation method. This study was registered with the PROSPERO (ID: CRD42020166174). In total, 16 studies including 10,605 participants (3913 case patients) were included. The pooled odds ratios (OR) and 95% confidence intervals (95% CI) for the highest versus the lowest 25(OH)D concentration was 0.39 (0.27, 0.57), with a high heterogeneity (*I*^2^ = 76.7%, *P* < 0.001). Meta-regression analysis identified latitude (*P* = 0.02), adjustment for gender (*P* = 0.001), and 25(OH)D stratification (*P* < 0.001) as sources of heterogeneity. Furthermore, the nonlinear dose–response analysis determined the OR (95% CI) of T1DM to be 0.91 (0.90, 0.93) per 10 nmol/L increase in the 25(OH)D concentration. A ‘U’-shaped association was found between serum 25(OH)D concentration and risk of T1DM. The present study highlights the significant inverse association between the circulating 25(OH)D concentration and the risk of T1DM.

## Introduction

Type 1 diabetes mellitus (T1DM) is an insulin-dependent diabetes that is characterized by immune-mediated destruction of pancreatic β cells, which leads to severe insulin deficiency [1]. The incidence of T1DM was 22.3/100,000 persons in the USA in 2014–2015, which has nearly doubled among the youth (aged <20 years) in the past decade [2]. A pooled analysis across 26 European centers recorded a 3.4%/annum increase in the incidence rate [3]. However, the estimated incidence of T1DM for individuals across ages is 1.01/100,000 persons in China [4]. It is believed that factors, such as obesity, breastfeeding, maternal and perinatal factors, virus infection, omega-3 fatty acid status, and serum 25(OH)D concentration play crucial roles in the prevention regimen of T1DM [1]. Reportedly, the number of patients with T1DM presenting with insufficient serum 25(OH)D concentration is growing rapidly [5].

The concentration of 25-hydroxy vitamin D [25(OH)D] in the serum reflects the status of vitamin D in the blood circulation [6]. Vitamin D deficiency has been identified to be common among patients with T1DM [5]. This secosteroid hormone is mainly produced from the precursor protein 7-dehydrocholesterol in the skin when exposed to solar ultraviolet B radiation and acquired slightly from the diet. The 25-hydroxylase enzyme in the liver transforms vitamin D to an intermediate inactive form of this vitamin, 25(OH)D, which is then transformed to the active form 1,25-hydroxy vitamin D by 1α-hydroxylase enzyme in the kidney [7]. As vitamin D regulates the immune system and autoimmunity, it may serve as a potential protective factor in the development of T1DM [1]. The effects of vitamin D on non-skeleton disorder remains debatable. Moreover, the role of serum 25(OH)D concentration in the risk of T1DM remains controversial [1, 7]. A birth-cohort study [8] in Finland suggested that sufficient vitamin D supplementation could assist in decreasing T1DM risk. A cross-sectional study [9] revealed that 70% of children with T1DM had vitamin D deficiency. The TEDDY study [10] reported that higher childhood 25(OH)D concentration is associated with lower islet autoimmunity, while prospective studies, such as DAISY and DIPP [11, 12] reported no such association between vitamin D intake or 25(OH)D concentration in the childhood and the risk of islet autoimmunity or T1DM. Moreover, vitamin D supplementation plays a protective role in hyperglycemia, while training anaerobically, and, in hypoglycemia, while training aerobically [13].

Several recent observational studies examined the relation between the serum 25(OH)D concentration and the risk of T1DM. Accordingly, we undertook the present meta-analysis to investigate the correlation between the serum 25(OH)D concentration and the risk of T1DM, while demonstrating their dose–response association.

## Methods

### Protocol and registration

This study was registered with the PROSPERO (ID: CRD42020166174).

### Search strategy and selection studies

The databases of PubMed, Embase, and Cochrane Library were searched for literatures published from inception to January 2020. Free-text terms and MeSH terms were used as follows: “type 1 diabetes” OR “type I diabetes” OR “insulin-dependent diabetes” OR “juvenile onset diabetes” OR “sudden onset diabetes” OR “autoimmune diabetes” OR “brittle diabetes” OR “Ketosis Prone diabetes” AND “vitamin D” OR “25-hydroxy vitamin D” OR “25(OH)D” OR “1,25-dihydroxy vitamin D” OR “1,25(OH)2D” OR “calcitriol” OR “calcidiol”. Potentially eligible studies were included without placing any language restriction in the search.

The following inclusion criteria were set to select clinical studies for the present meta-analysis: (1) Observational studies on humans, except for cross-sectional studies; (2) All subjects met the diagnosis criteria of T1DM; (3) The serum 25(OH)D concentration was measured quantitatively; (4) Studies that evaluated the association between the 25(OH)D concentration and the risk of T1DM and were obliged to report the odds ratios (OR), relative risk or hazard ratios with the corresponding 95% confidence intervals (95% CI) or useful data for these statistics. All reviews, case reports, letters, registration of trials, and conference abstracts without full-text link were excluded from the study.

### Data extraction and quality assessment

**Fig. 1:**
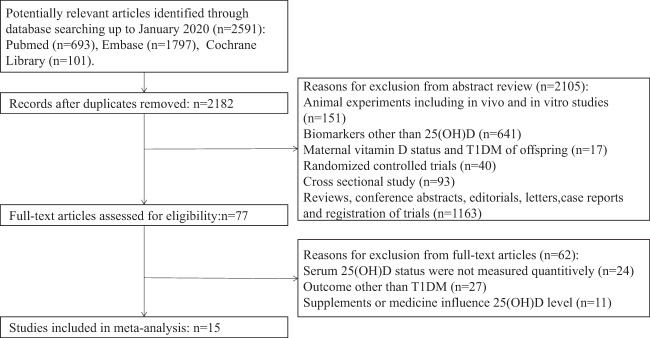
The flow diagram of literature research and study selection.

All literatures collected according to the search strategy were independently assessed by two authors (YH and YJ), who were blinded to author and journal details, to identify potentially eligible studies. Any issue that presented was addressed by discussion with the third author, GS. The literature selection method flowchart is illustrated in Fig. 1. The following data were extracted from the included studies: the name of the first author, publication year, study design, geographical locations (based on latitude search on Google Maps), age, gender, body mass index, risk of T1DM, serum 25(OH)D concentration, and assay method for 25(OH)D concentration estimation. Quality assessment was gauged with reference to the Newcastle–Ottawa Scale for cohort and case-control studies [14]. A score of 4–6 was considered moderate quality and that of 7–9 as high quality.

### Statistical analysis

OR and 95% CI were obtained as the summary risk estimates for T1DM from all studies, and the relative risks and the hazard ratios were esteemed to be equivalent to OR. Heterogeneity among the studies was estimated by Cochrane Q test and *I*^2^ statistic, which was confirmed to be statistically significant at *P* < 0.05 and *I*^2^ > 50%. Pooled OR and 95% CI were evaluated using the random-effects model (DerSimonian and Laird method) [15]. To scrutinize the potential source of heterogeneity, manifold subgroup analysis ranging from latitude of geographical locations, serum 25(OH)D concentration, and assay method for estimating the 25(OH)D concentration were performed. Heterogeneity between the subgroups was measured by meta-regression analysis. At *P* < 0.05 for meta-regression, the heterogeneity between the subgroups was considered to be significant [16]. By omitting one study at a time, we conducted a sensitivity analysis to test for the robustness of the pooled results. Forest plots were applied to describe the summary effects. Publication bias was separately assessed via funnel plot and Egger’s test. The funnel plot was asymmetrical or the *P* < 0.10 was for Egger’s test, indicating potential publication bias [17]. In addition, the overall effects were estimated by the trim and fill method after adjusting for the missing studies [15, 18].

Based on the dissimilar cut-off points for categories in individual article, we computed OR and 95% CI of T1DM for every 10 nmol/L of serum 25(OH)D concentration increase via generalized least squares for trend estimation, as suggested by Greenland and Longnecker [19] and Orsini [20]. The mean concentration of the serum 25(OH)D concentration in each category was considered as the corresponding dose. If the boundary of the highest category was open ended, the midpoint of this category was set at the lower boundary, multiplied by 1.5. If the lowest category was unavailable, we set it as zero. The 25(OH)D concentration and T1DM risk for each distribution of cases and controls were distilled as per the method. In this dose–response analysis, we excluded studies that did not quantify the number of cases and controls per category as well as those that reported 25(OH)D concentration with OR and 95% CI for less than 3 categories.

In addition, we appraised a potential curve for dose–response relationship between the 25(OH)D concentration and the summary OR and 95% CI for T1DM. A restricted cubic spline model with four knots at the 5th, 35th, 65th, and 95th percentiles of 25(OH)D concentration was used. The linear and nonlinear models were calculated by testing the null hypothesis, with the splines coefficient considered zero.

All statistical analyses were performed using the Stata version 15.1.614 (Stata Corp, Texas, USA).

## Results

### Literature research and study characteristics

Based on our initial literature research, 2591 articles were identified, of which 409 that were identified to be duplicates and 2105 that were uncorrelated, were removed. After the full-text screening, 62 articles were excluded as they did not meet all of the eligible criteria listed in Fig. 1. Finally, 12 case-control studies [21–32], two nested case-control studies [33, 34], one case-cohort study [28], and one cross-sectional case-control study [35] were pooled in the meta-analysis.

The characteristics of each included studies are outlined in Table 1. Briefly, 10,605 subjects with 3913 T1DM cases and 6692 healthy controls participated in the 16 studies. The geographical locations of all the study areas were based in the Northern Hemisphere. With respect to the age groups, 12 studies were conducted among children [21, 22, 24–29, 31, 32, 35], two in adults [33, 34], and two in a blended population of children and young adults [23, 30]. Gorham et al. [33] and Munger et al. [34] conducted their studies among military service members of the United States. When assessed with reference to the Newcastle–Ottawa Scale, five studies [25–28, 31] showed a moderate quality score and 11 [21–24, 28–30, 32–35] showed a high-quality score. The characteristics of the serum 25(OH)D concentration and the risk of T1DM are detailed in Table 2. The OR and 95% CI were classified by each category of 25(OH)D concentration from 5.14 to 250 nmol/L. Various assay methods were applied to quantify the 25(OH)D concentration. Most matched or adjustment variables were identified to be age, gender, and ethnic traits. The duration of T1DM ranged from being newly diagnosed to having been diagnosed for several years.

**Table 1:** Characteristics of the included studies in the meta-analysis.

Author, year	Country	Study design	Geographic Location		NOS	Participants			
			Region	Latitude, °N		Ethnicity, case/control	No. of gender, male/female	Age of cases/controls, year	BMI of cases/controls, kg/m^2^
Bener [21]	Qatar	case-control study	Doha	25	8	White, wheat, brown or black people	168/172	10.5 ± 3.8/9.9 ± 4.2	16.76/17.88
Borkar [35]	India	cross-sectional case-control study	Chandigarh	31	8	NA	55/45	8.63 ± 2.01/8.48 ± 1.58	15.74 ± 1.88/16.25 ± 2.43
Hamed [22]	India	case-control study	Assuit	27	7	NA	20/31	10.38 ± 3.17/8.47 ± 4.17	17.83 ± 7.07/16.42 ± 2.88
Gorham [33]	USA	nested case-control study	San Diego	32	7	White, black, and other	NA	33.28/33.26	NA
Ghandchi [23]	Iran	case-control study	Tehran	35	8	NA	58/62	14.4(0.5)/14.6(0.8)^a^	21.4(0.6)/20.8(0.7)^a^
Munger [34]	USA	nested case-control study	Boston	42	7	Non-Hispanic Whites	439/23	20.6 ± 4.1/20.6 ± 4.0	NA
Franchi [26]	Italy	case-control study	Verona	45	4	Caucasian and other	124/100	9.2(1.1–16.0)/8.7(1.1–16.3)^b^	16.6(11.4–25.8)/16.9(11.0–38.3)^b^
Azab [24]	Egypt	case-control study	Zagazig	30	7	NA	58/62	11.4 ± 2.5/10.8 ± 2.3	23.6 ± 5.7/21.8 ± 4.7
Abd-Allah [25]	Egypt	case-control study	Zagazig	30	6	NA	90/150	11.7 ± 2.8/11.1 ± 2.6	18.5 ± 4.3/21.6 ± 1.5
Cadario [27]	Italy	case-control study	Novara	45	6	Italian, Northern African, and Eastern European	145/158	7.0 ± 0.25/7.2 ± 0.49	NA
Jacobsen [28]	Denmark	case-cohort study	Denmark	55	6	Danish, other western, other non-western	1933/1852	10.2(7.5,12.5)/10.5(5.1,11.5)^c^	NA
Jacobsen [28]	Denmark	case-control study	Denmark	55	9	Danish, other western, other non-western	551/503	8.5(5.0,22.4)/NA^c^	NA
Rasoul [29]	Kuwait	case-control study	Safat	29	8	Kuwaiti Arab	210/210	9.59 ± 3.20/9.60 ± 2.73	NA
Bae [30]	Korea	case-control study	Seoul	37	9	Korean	276/327	14.5 ± 4.4/13.8 ± 3.8	SDS: −0.01 ± 0.95/−0.09 ± 1.19
Liu [32]	China	case-control study	Nanjing	32	8	Chinese	295/296	8.66 ± 3.75/8.40 ± 3.60	NA
Federico [31]	Italy	case-control study	Varese, Pisa	45	5	NA	107/92	9.5 ± 3.8(5.0)/9.4 ± 3.9(6.5)^d^	NA

*T1DM* type 1 diabetes mellitus, *NOS* Newcastle–Ottawa Scale, *BMI* body mass index, *NA* not available, *N* number, *SDS* standard deviation score.

^a^Age and BMI were presented as mean ± standard deviation except a was mean (standard error).

^b^Median, full range (min–max).

^c^Median (interquartile range).

^d^Median ± standard deviation (interquartile range).

**Table 2:** Characteristics of categories of serum 25(OH)D concentration and risk of T1DM.

Author, year	Cat. of vitamin D, nmol/L	Number (case/control)	Risk of T1DM		Assay method of 25(OH)D	Matched or adjustment variables	Duration of T1DM
			OR	95% CI			
Bener [21]	<75	154/145	1	(reference)	RIA	Age, gender, and ethnicity of case	NA
	75–200	16/25	0.6	(0.31, 1.17)			
Borkar [35]	<50	29/16	1	(reference)	HPLC	Age, gender	The cases were diagnosed within 1 week.
	50–75	14/22	0.35	(0.14, 0.87)			
	>75	7/12	0.32	(0.11, 0.98)			
Hamed [22]	<50	19/0	1	(reference)	CPBA	Age, pubertal stage, and physical activity	The duration of T1DM was 2.67 ± 3.52 years.
	>50	17/15	0.03	(0, 0.52)			
Gorham [33]	<43	52/15	1	(reference)	CLIA	The date that the blood sample was drawn, age, length of military service, gender, and whether the control was on active duty when the case was diagnosed	NA
	43–59	50/20	0.72	(0.33, 1.56)			
	60–77	33/26	0.37	(0.17, 0.79)			
	78–99	34/32	0.31	(0.14, 0.65)			
	≥100	933/805	0.33	(0.19, 0.6)			
Ghandchi [23]	<28	51/50	1	(reference)	HPLC	Siblings at the same age	The duration of T1DM for anti-GAD-Ab+ cases was 3.1 ± 2.4 years while for anti-GAD-Ab- cases was 6.6 ± 4.8 years.
	28–50	6/4	1.47	(0.39, 5.53)			
	≥50	3/6	0.49	(0.12, 2.07)			
Munger [34]	31–77	47/50	1	(reference)	CLIA	Age, gender, race/ethnicity, dates of serum collection, and branch of military service	NA
	77–89	30/62	0.52	(0.28, 0.93)			
	89–101	29/66	0.47	(0.26, 0.84)			
	101–114	19/66	0.31	(0.16, 0.59)			
	114–211	29/64	0.48	(0.27, 0.87)			
Franchi [26]	≤50	39/86	1	(reference)	CLIA	Age, gender, and season of hospitalization	All cases were newly diagnosed.
	51–74	14/44	0.7	(0.35, 1.43)			
	≥75	5/36	0.31	(0.11, 0.84)			
Azab [24]	<50	44/12	1	(reference)	ELISA	Age and gender	The median (min–max) of duration of T1DM was 17(3–52) months.
	>50	36/28	0.35	(0.16, 0.79)			
Abd-Allah [25]	<38	84/36	1	(reference)	ELISA	Age, gender, and ethnic origin	NA
	38–50	6/18	0.41	(0.05, 0.39)			
	>50	30/66	0.2	(0.11, 0.35)			
Cadario [27]	<5	36/103	1	(reference)	LC-MS	Birthday, place of birth, and ethnic group	The age at diagnosis was 4.2 ± 3.4 years old.
	≥5	31/133	0.67	(0.39, 1.15)			
Jacobsen [28]^a,b^	0–12	NA	1	(reference)	LC-MS	NA	The age of onset was 10.2(7.5,12.5) for 912 cases and 10.5(5.1,11.5) for 7 cases in cohort.
	12–20	NA	1.13	(0.80, 1.58)			
	20–28	NA	1.16	(0.90, 1.50)			
	28–41	NA	1.01	(0.72, 1.42)			
	41–130	NA	1.13	(0.81, 1.57)			
Jacobsen [28]^b^	0–11	NA	1	(reference)	LC-MS	Date and season of birth	The age of onset was 8.5(5.0,11.4).
	11–17	NA	1.66	(0.69, 3.99)			
	17–25	NA	1.61	(0.84, 3.09)			
	25–36	NA	1.32	(0.56, 3.12)			
	36–118	NA	1.32	(0.55, 3.18)			
Rasoul [29]	<53	182/158	1	(reference)	ELISA	Age, gender, and ethnicity	The onset age of T1DM was <4 years in 20% patients, 4–6 years in 28% patients and >6 years in 52% patients.
	53–73	31/29	0.93	(0.54, 1.61)			
	>73	3/11	0.24	(0.07, 0.86)			
Bae [30]	<50	41/136	1	(reference)	RIA	Age, gender, and body mass index	4.6 ± 4.0 years for 25(OH)D < 48nmol/L; 5.4 ± 3.4 for 25(OH)D 48–72nmol/L; 2.4 ± 2.4 for 25(OH)D ≥72 nmol/L
	50–75	30/206	0.48	(0.29, 0.81)			
	≥75	14/176	0.26	(0.14, 0.50)			
Liu [32]	<30	39/17	1	(reference)	ELISA	Age, gender, and ethnicity	106 cases were diagnosed not longer than 30 days, while 190 cases were diagnosed longer than 30 days.
	30–50	108/73	0.65	(0.34, 1.23)			
	>50	149/205	0.32	(0.17, 0.58)			
Federico [31]	≤50	41/20	1	(reference)	HPLC	Age, gender	The age at the clinical onset of T1DM was 9.4 ± 3.9 years (range 2.1–1.8).
	50–75	21/36	0.28	(0.13, 0.61)			
	75–250	20/61	0.16	(0.08, 0.33)			

Duration of T1DM was presented as mean ± standard deviation.

*T1DM* type 1 diabetes mellitus, *25(OH)D* 25-hydroxy vitamin D, *Cat*. categories, *OR* odds ratio, *95% CI* 95% confidential interval, *NA* not available, *RIA* radioimmunoassay, *ELISA* enzyme-linked immunosorbent assay, *LC-MS* liquid chromatography coupled with mass spectrometry, *HPLC* high-performance liquid chromatography, *CLIA* chemiluminescence immunoassay, *CPBA* competitive binding protein assay, *NOS* Newcastle–Ottawa Scale, *BMI* body mass index, *NA* not available, *N* number, *SDS* standard deviation score.

^a^We used hazard ratios to present the risk of T1DM.

^b^The duration of T1DM was presented as median (interquartile range).

### Meta-analysis

**Fig. 2:**
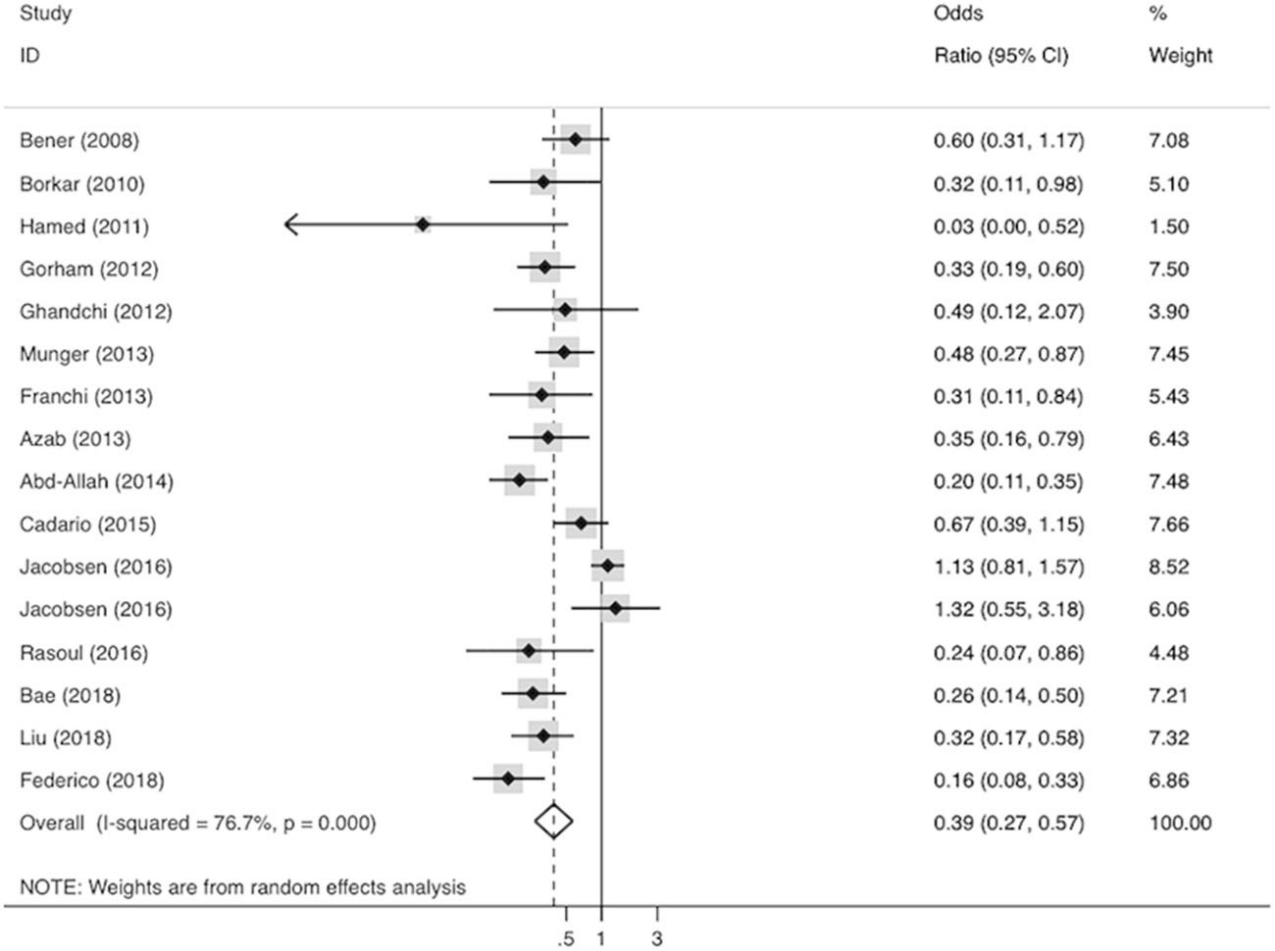
Forest plot for pooled effects of serum 25(OH)D concentration on risk of T1DM.

The forest plot for the pooled effects of the highest versus the lowest cut-off point of 25(OH)D concentration on the risk of T1DM is illustrated in Fig. 2. The case-cohort study and the case-control study examined by Jacobsen et al. [28] did not provide eligible number of cases and controls for each category. Moreover, they did not use the lowest cut-off point of 25(OH)D concentration as the reference, therefore, we transformed the OR and 95% CI extracted from them into the lowest cut-off point as a reference, based on the method introduced by Hamling et al. [36]. The hazard ratios of the case-cohort study were deemed as OR. The summary OR (95% CI) was 0.39 (0.27, 0.57) for the highest cut-off point as compared with the lowest cut-off point of 25(OH)D concentration. The *P* value of Cochrane Q test was <0.001, and the *I*^2^ statistics was 76.7%, which indicated that the heterogeneity among the analyzed studies was significant.

### Subgroup, meta-regression, and sensitivity analysis

**Fig. 3:**
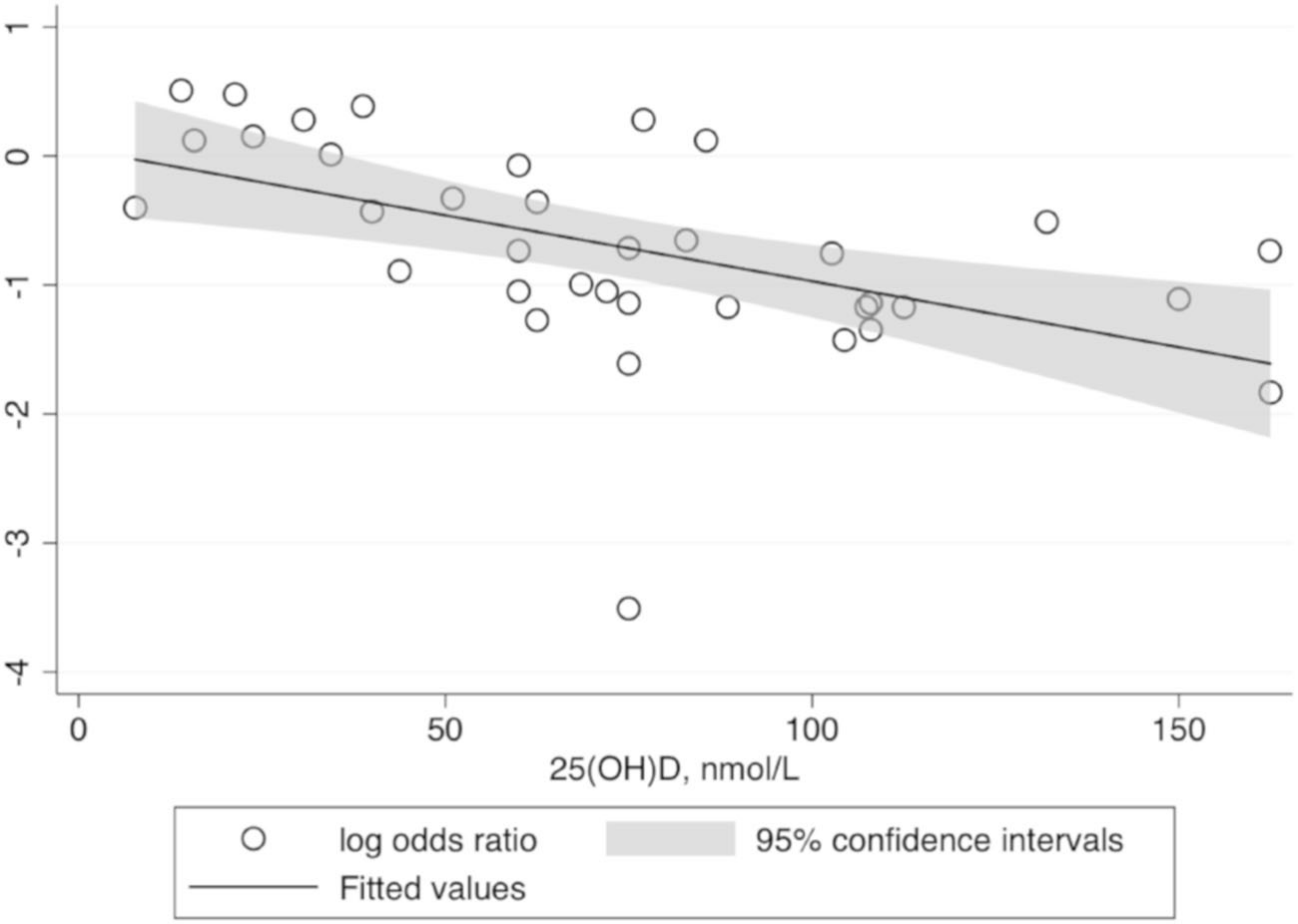
Meta-regression for all categories of 25(OH)D concentration and risk of T1DM.

**Fig. 4:**
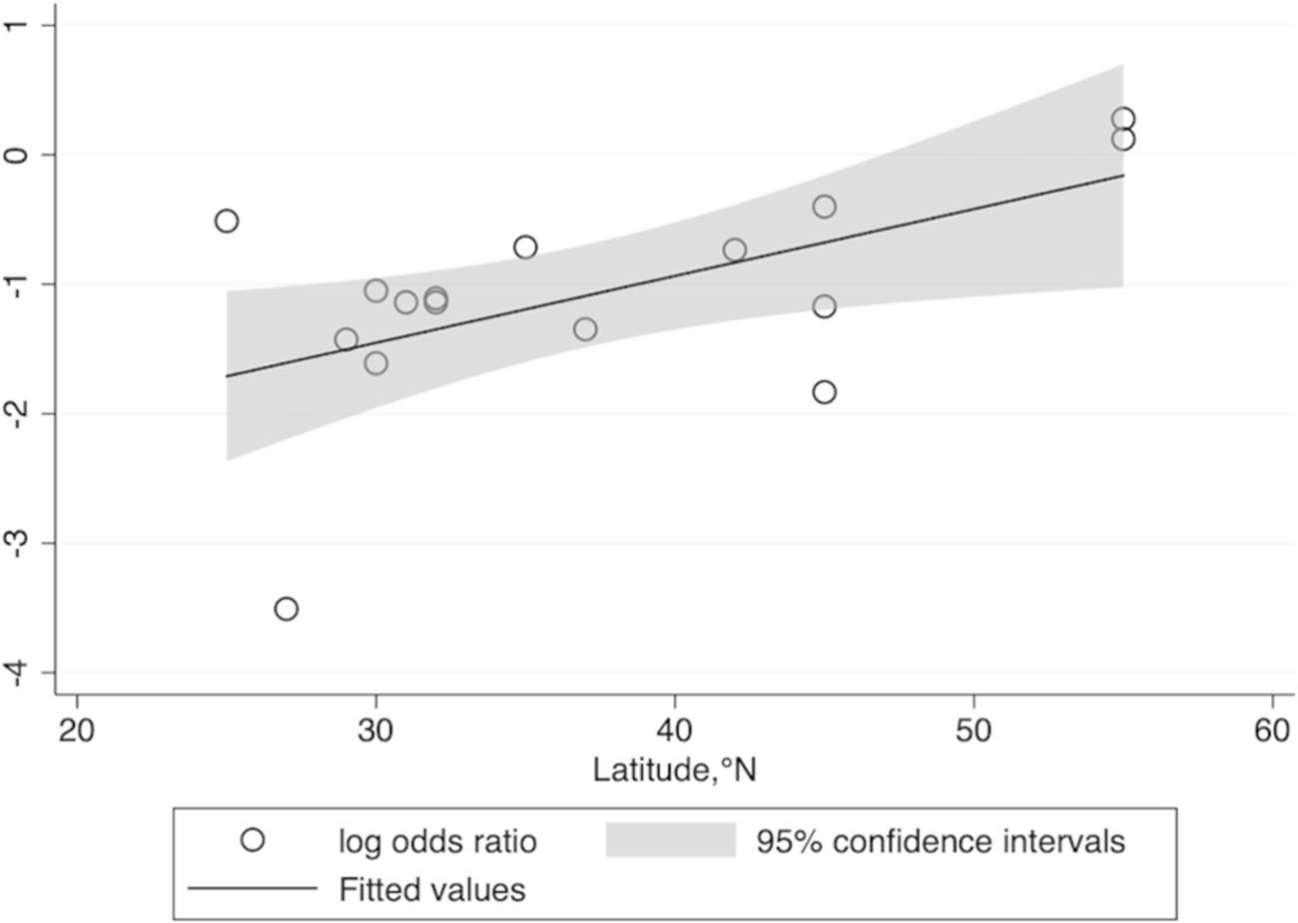
Meta-regression for latitude and risk of T1DM.

Subgroup analysis and meta-regression are summarized in Table 3. Meta-regression was exerted to explore the heterogeneity among the subgroups. All categories of 25(OH)D concentration extracted from the 16 studies were included in this meta-regression. The results of the analysis revealed negative correlation between the serum 25(OH)D concentration and log OR of T1DM with statistical significance (*P*_between_ < 0.001; Fig. 3). Latitude for each study was also considered, with the results showing positive correlation with statistical significance (*P*_between_ = 0.02; Fig. 4). Adjustment for gender indicated statistical significance (*P*_between_ = 0.001) between the subgroups. Nevertheless, the subgroups stratified by age, ethnicity, assay method for estimating 25(OH)D concentration, duration of T1DM, and adjustment of ethnicity showed no statistical significance between the subgroups in heterogeneity.

**Table 3:** Subgroup analysis for risk of T1DM.

Subgroup	No. of studies	Risk of T1DM^a^		I^2^, %	*P*_within_^b^	*P*_between_^c^
		OR	95% CI			
Overall	16	0.39	(0.27, 0.57)	76.70%		
Age						
Only children	12	0.40	(0.24, 0.64)	81.1	<0.001	0.77
Other	4	0.36	(0.26, 0.50)	0.0	0.53	
Ethnicity						
White	2	0.42	(0.25, 0.72)	0.0	0.33	0.16
Yellow	2	0.29	(0.19, 0.45)	0.0	0.65	
Mix	6	0.65	(0.4, 1.05)	73.4	0.002	
Latitude, °N						
≤30	5	0.29	(0.16, 0.54)	55.3	0.06	0.02
31–45	9	0.35	(0.26, 0.47)	36.4	0.13	
>45	2	1.15	(0.85, 1.57)	0.0	0.75	
Categories of 25(OH)D, nmol/L						
≤50	1	0.67	(0.39, 1.15)	NA	NA	< 0.001
50–75	5	0.27	(0.18, 0.41)	16.8	0.31	
75–100	2	1.15	(0.85, 1.57)	0.0	0.75	
>100	8	0.33	(0.24, 0.45)	26.3	0.22	
Assay method of 25(OH)D						
RIA	2	0.39	(0.17, 0.89)	68.5	0.08	0.10
HPLC	3	0.24	(0.13, 0.45)	17.9	0.30	
CPBA	1	0.03	(0.00, 0.48)	NA	NA	
CLIA	3	0.38	(0.26, 0.56)	0.0	0.61	
ELISA	4	0.27	(0.19, 0.38)	0.0	0.62	
LC-MS	3	0.98	(0.68, 1.42)	33.6	0.22	
Duration of T1DM						
>1year	3	0.25	(0.13, 0.47)	17.3	0.30	0.50
<1year	2	0.38	(0.16, 0.89)	0.0	0.64	
Adjustment for ethnicity						
Yes	6	0.40	(0.26, 0.60)	58.1	0.04	0.98
No	10	0.38	(0.22, 0.68)	81.9	<0.001	
Adjustment for gender						
Yes	11	0.31	(0.25, 0.39)	17.3	0.28	0.001
No	5	0.80	(0.46, 1.39)	59.8	0.04	

*T1DM* type 1 diabetes, *N* number, *OR* odds ratio, *95% CI* 95% confidential interval.

^a^Summary OR and 95%CI were calculated by random-effects models.

^b^*P*_within_ was the *P* value for heterogeneity within each subgroup.

^c^*P*_between_ calculated by meta-regression was the *P* value for heterogeneity between subgroups

Sensitivity analysis was conducted to investigate which individual study with extreme OR influenced the pooled OR. For instance, if the study by Hamed et al. [22] with the smallest sample was omitted, the summary effects changed to summary OR (95% CI) 0.41 (0.28, 0.59). Moreover, if the case-cohort study and case-control study published by Jacobson et al. [28] with the relatively largest sample size were omitted, the summary effects changed to summary OR (95% CI) 0.36 (0.27, 0.48) and summary OR (95% CI) 0.36 (0.25, 0.53), respectively. However, the summary effects were not influenced substantially.

### Dose–response meta-analysis

**Fig. 5:**
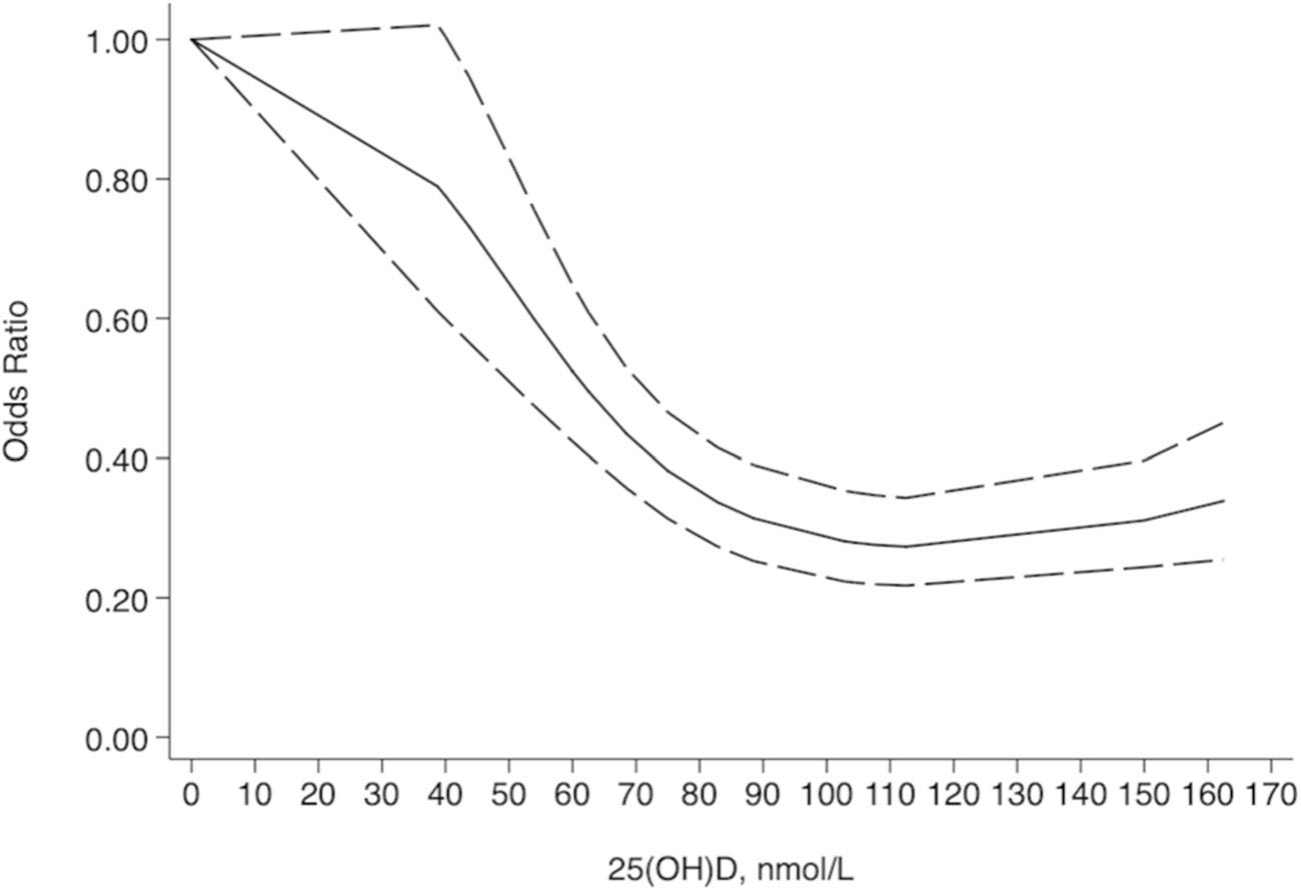
Dose–response analysis of serum 25(OH)D concentration and risk of T1DM.

Figure 5 displays the results of dose–response analysis. A total of 10 studies with 2223 cases and 2730 controls were involved in the dose–response meta-analysis. Studies by Bener et al. [21], Hamed et al. [22], Azab et al. [24], and Cadario et al. [27] were excluded from the dose–response meta-analysis, considering that they did not stratify the serum 25(OH)D concentration in not less than three categories. Similarly, the two studies by Jacobsen et al. [28] were also excluded because they did not provide the number of cases and controls for each category.

We noted an inverse nonlinear association between the serum 25(OH)D concentration and the T1DM risk (chi-square = 131.08, *P* < 0.001), and the heterogeneity was insignificant (*Q* = 22.24, *P* = 0.39) for both fixed and random models. The OR (95% CI) of T1DM was 0.91 (0.90, 0.93) per 10 nmol/L increase in the 25(OH)D concentration. A ‘U’-shaped association was found. The risk of T1DM significantly descended with the 25(OH)D concentration ranging from 39 to 89 nmol/L with OR (95% CI) from 0.79 (0.61, 1.02) to 0.31 (0.25, 0.39), trended stably when the 25(OH)D concentration reached 103–113 nmol/L with OR (95% CI) around 0.28 (0.22, 0.35), and slightly ascended when the 25(OH)D concentration surpassed 150 nmol/L with OR 95% CI and 0.34 (0.25, 0.45).

### Publication bias

The funnel plot (Supplementary Material File) was asymmetrical and the Egger’s test revealed *P* = 0.03, suggesting that publication bias was significant among the studies. Nevertheless, the results did not fluctuate after a trim and fill test, indicating that the impact of the publication bias was probably modest.

## Discussion

In this meta-analysis of 16 observational studies including 10,605 participants, a significant inverse association was recorded between the highest versus the lowest circulating 25(OH)D concentration and the T1DM risk. This analysis demonstrated the role of vitamin D as a protective factor for T1DM, albeit the heterogeneity among the pooled studies was significant. In support, Shen et al. [37] reported that patients with T1DM showed a lower 25(OH)D concentration than controls in a meta-analysis of 12 studies including 3885 participants. Furthermore, Rak et al. [38] asserted that proper vitamin D supplementation could reduce the incidence and complications of T1DM.

Subgroup analysis along with meta-regression was applied to explore the source of heterogeneity. We recognized a positive association between the latitude of patient location and risk of T1DM, which suggests that individuals living in high latitude may be predisposed to T1DM. Weng et al. [4] also reported that the incidence of T1DM among children aged <14 years was strongly correlated with the latitude, with the rates being higher in the north of China than in the south. Kimlin et al. [39] emphasized that the latitude strongly influences the serum 25(OH)D concentration among participants across a broad latitude range in the southern hemisphere. Mohr et al. [40] identified that the incidence of T1DM tended to be higher at higher latitudes in both the hemispheres, because residents living near the Equator obtained adequate vitamin D due to the strong solar ultraviolet B irradiance available there. More consistent studies indicated that the latitude influences the serum 25(OH)D concentration, which in turn affects the incidence rate of T1DM.

Adjustment for gender was identified as a potential source of heterogeneity. A previous study EURODIAB found that the incidence of T1DM was nearly the same between both the genders of children [41]. However, Weng et al. [4] discovered that the incidence of T1DM was higher among girls under the age of 14 years. Thus, adjustment for gender among individual studies needs to be carefully evaluated in the future.

We also identified that the association was undeviating and did not diverge appreciably after stratifying by age, ethnicity, assay method for estimating the 25(OH)D concentration, duration of T1DM, and adjustment for ethnicity. Shen et al. [37] found that the 25(OH)D concentration was lower in patients with T1DM than in controls in a subgroup population aged ≤14 years. High-performance liquid chromatography (HPLC) was considered as the golden standard assay method to determine the 25(OH)D concentration [42]. Al-Haddad et al. [43] emphasized that chemiluminescence micro-particle immunoassay overestimates vitamin D deficiency in comparison to HPLC. These reports suggest that physicians should select the assay method for estimating the 25(OH)D concentration extremely cautiously. However, in our study, the pooled OR of T1DM did not differ by the assay methods. This difference from other reports may be attributed to statistical fluctuation in the small sample size in this study, highlighting the need for further studies to clarify the source of heterogeneity.

Notably, we observed a significant inverse correlation between the circulating 25(OH)D concentration and the risk of T1DM and developed a robust database supporting the dose–response curve. To the best of our knowledge, no previous studies have addressed the quantitative association between serum 25(OH)D concentration of 100–150 nmol/L to the significantly lower risk of T1DM. Functionally, sufficient serum 25(OH)D concentration can preserve the activity of residual pancreatic β-cells and insulin secretion [44]. Meanwhile, a past study reported that the serum 25(OH)D concentration was negatively associated with insulin resistance in patients with T1DM [45]. Children with T1DM have also been reported to have lower 25(OH)D concentration than healthy children [5]. Low 25(OH)D concentration may be associated with lower insulin concentration in hepatic portal vein inhibiting the 25-hydroxylase activity. Although intraperitoneal insulin increased the 25(OH)D concentration in the hepatic vein, long-term intraperitoneal insulin treatment did not affect the 25(OH)D concentration when compared with subcutaneous insulin treatment in patients with T1DM [46]. Moreover, from the perspective of the Nutrition Society, the guidelines for vitamin D supplementation and assay standard of 25(OH)D concentration are in a standstill period [47]. The nutritional guideline from Netherlands [48] suggests that a serum 25(OH)D concentration of >30 nmol/L is sufficient for individuals except for adults above the age of 70 years whose target serum 25(OH)D concentration is beyond 50 nmol/L. Besides, the Scientific Advisory Committee on Nutrition [49] and German Nutrition Society [50] advocate that serum 25(OH)D concentration maintains at least 50 nmol/L for generally healthy individuals. However, the German vitamin D intake recommendations (200–800 IU per day) had no effect on improving the serum 25(OH)D concentration of infants and adolescents in the latest report, which means they need to revise the guideline [51]. The endocrine guideline from the Middle East and North Africa [52] also recommends a serum 25(OH)D concentration of >50 nmol/L is sufficient for generally healthy individuals, while the Endocrine Society [53] supports serum 25(OH)D concentration of >75 nmol/L for individuals in all ages including pregnancy and lactating. Other guidelines from Australia [54] uphold that serum 25(OH)D concentration of >50 nmol/L is sufficient for pregnant women and the National Osteoporosis Foundation [55] and the bone-centric guidelines [6] support serum 25(OH)D concentration of >50 nmol/L is adequate for nearly the entire population, while American Geriatrics Society [56] proposes serum 25(OH)D concentration of >75 nmol/L is sufficient for adults aged over 70 years. Moreover, the guidelines focused on the pleiotropic effects of vitamin D recommended a target 25(OH)D concentration of 75 nmol/L [6]. Thus, guidelines for target serum 25(OH)D concentration from nutritional and endocrine fields differ, probably because they focused on different populations and geographic locations. Interestingly, we found that most guidelines targeted adults aged beyond 70 years set their goal for vitamin D sufficiency is 75 nmol/L which is higher than others because the elder is more likely to fall and fracture. For infants, children, pregnant or lactating women, most guidelines agree with optimal serum 25(OH)D concentration of >50 nmol/L or depending on their health condition. Notably, most European vitamin D researchers and organizations considered serum 25(OH)D concentration 50 nmol/L to be necessary, while in the United States, they recommend 75 nmol/L and some others are calling for 100–150 nmol/L. The difference between the recommendations from the two continents may be due to the greater reliance on RCTs by European researches. Unfortunately, most RCTs up to now were poorly designed and conducted since they were based on guidelines for drugs, not nutrients [57]. So, the RCT designers should pay more attention to serum 25(OH)D concentration, not vitamin D dose [58]. What is more, the relationship between serum 25(OH)D concentration and health outcomes may vary for different diseases [6]. The viewpoints from the Scientific Advisory Committee on Nutrition [49], Middle East and North Africa [52] and the Endocrine Society [53] espouse that vitamin D is a protective factor for diabetes, while others show uncertainty. This meta-analysis has explored the connection between them.

Our meta-analysis identified contradicting results with those from past RCTs. The daily supplementation of vitamin D differs across countries and organizations. For children under 18 years, the recommendation vitamin D dose was 200–1000 IU [59]. And it depended on age, health status, body weight, and race that the daily vitamin D dose recommendation ranged from 400–2000 IU [6]. Therefore, no consensus has yet been reached regarding the ideal daily supplementation concentration. Shih et al. [60] conducted a randomized prospective crossover study with 25 adolescents with T1DM and found that even a 6-month-long vitamin D repletion did not affect the status of glycemia or inflammatory biomarkers. Similarly, Sharma et al. [61] reported that the administration of oral vitamin D therapy once a month for 6 months led to no significant decrease in the HbA1c status and in the exogenous insulin requirements in their double-blinded RCT including 52 children. Recently, Kadhim et al. [62] conducted an RCT, wherein they provided 50 newly diagnosed pediatric patients and 25 healthy children with a daily vitamin D3 dosage of 2000 IU for a period of 90 days; the authors recorded significantly positive immune response and an increase in the serum 25(OH)D concentration for the patients. Previous RCTs were majorly conducted in participants without insufficient serum 25(OH)D concentration. Most meta-analyses resulting from RCT did not reveal that the vitamin D supplementation benefited health, which contradicts the observational studies, which claim that insufficient serum 25(OH)D concentration has adverse ill effects on health [63]. Owing to the fixed vitamin D dose in the interventional studies, the circulating 25(OH)D concentration may have fluctuated in a narrow range, which could not build a substantial cause–effect association between vitamin D supplementation and positive health outcomes. Our meta-analysis suggests that, in the future, studies should set a target of serum 25(OH)D concentration when supplying vitamin D for yielding stable results.

Notwithstanding, we noted some limitations in our study. For instance, most of the included articles were case-control studies, which is not a relatively robust study design toward confirming a causal relationship. As compared with studies that record data in the real time, case-control studies have a greater probability of recall bias. In addition, this meta-analysis was based on observational studies that were generally not considered to be able to demonstrate causality between serum 25(OH)D concentration and T1DM risk [64]. However, according to Hill’s criteria for causality, we disclosed the dose–response curve that cast a new light on the possible causality that low serum 25(OH)D concentration had adverse effects on T1DM [65, 66].

## Conclusions

In conclusion, through our dose–response meta-analysis enlisting 16 studies, we demonstrated a significant inverse association between the 25(OH)D concentration in circulation and the risk of T1DM. The resultant dose–response relationship also provided a broad 25(OH)D spectrum. We suggest the necessity for further studies focusing on the molecular mechanism underlying this association. In clinical relevance, optimized and well-designed RCT are necessary to yield greater insights to the benefits and safety of vitamin D supplementation in preventing the risk of developing T1DM.

Supplementary information is available at EJCN’s website.

## Supplementary information

Supplementary Material File
