# Supplementary figures and images for: A dose–response meta-analysis between serum concentration of 25-hydroxy vitamin D and risk of type 1 diabetes mellitus
Source: Eur J Clin Nutr. 2020 Nov 24;75(7):1010–23. doi: 10.1038/s41430-020-00813-1 (PMC8266682; doi:10.1038/s41430-020-00813-1)

Funnel plot with pseudo 95% confidence limits

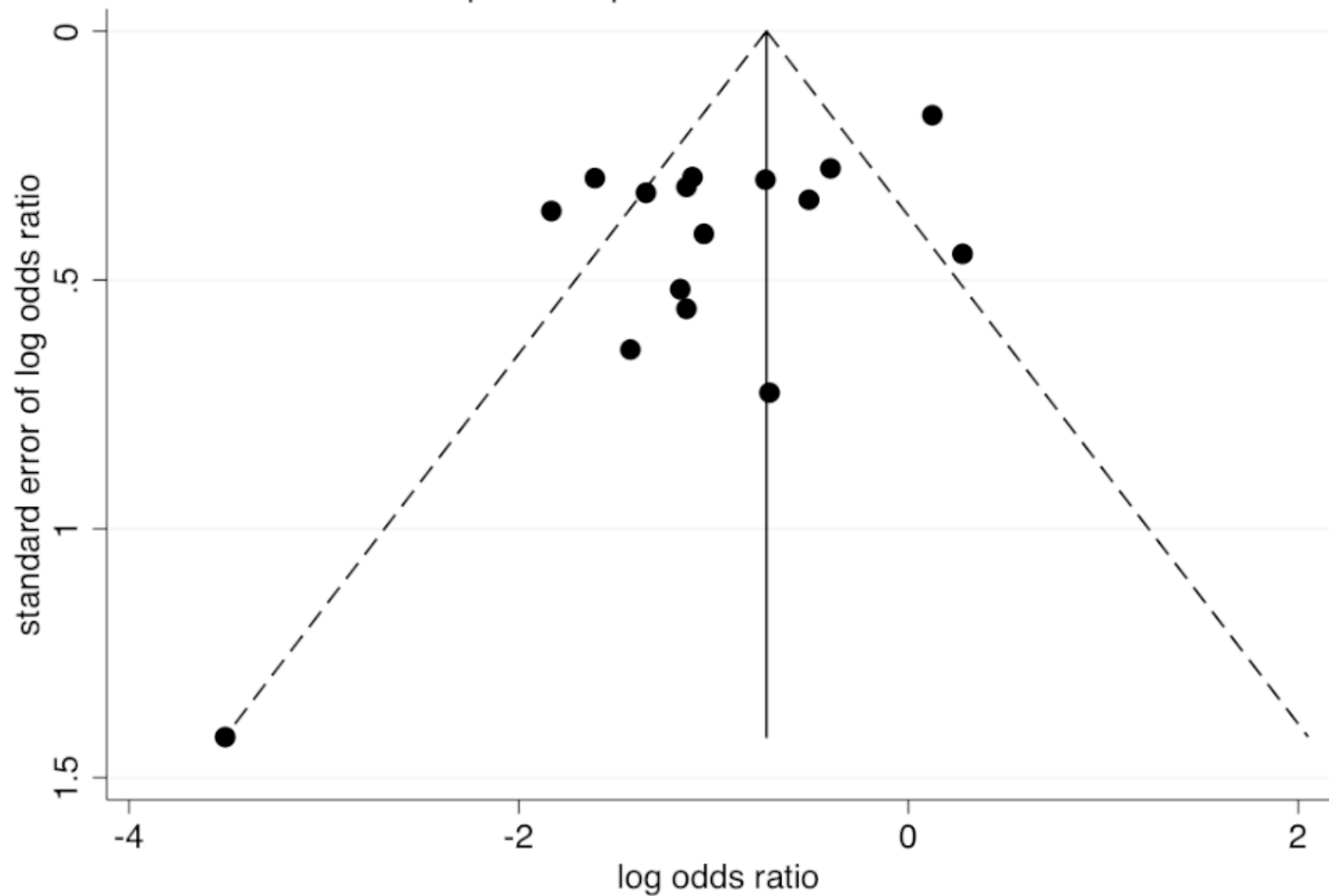

Supplement: Supplementary file 1 — Supplementary Material File [file 41430_2020_813_MOESM1_ESM.pdf]
